# “Exercise… to Me, It’s Freedom”: Motivation, Support, and Self-Management to Keep Physically Active with Parkinson’s Disease: A Qualitative Study

**DOI:** 10.3390/geriatrics9040092

**Published:** 2024-07-11

**Authors:** Leanne Ahern, Catriona Curtin, Suzanne Timmons, Sarah E. Lamb, Ruth McCullagh

**Affiliations:** 1 Discipline of Physiotherapy, School of Clinical Therapies, University College Cork, T12 X70A Cork, Ireland; 2 Centre for Gerontology and Rehabilitation, School of Medicine, College of Medicine and Health, University College Cork, T12 FN70 Cork, Ireland; 3 Faculty of Health and Life Sciences, University of Exeter, Exeter EX1 2LU, UK

**Keywords:** Parkinson’s Disease, exercise, physiotherapists, social stigma, qualitative research

## Abstract

The benefits of exercise have been widely explored among people with Parkinson’s (PwP). Exercise can improve non-motor (fatigue, pain, sleep, etc.) and motor features (balance, muscle strength, gait speed, etc.), maintain function, as well as prevent disease progression. Although the benefits are well known, PwP continue to show difficulty adhering to physical activity and exercise. This study aims to explore motivation to exercise, support, and self-management needs among people with Parkinson’s, their family members, and physiotherapists. Purposeful and maximum-variation sampling methods (age, sex, geographical setting, and disease severity) were employed. PwP and family members were recruited through physiotherapy services and local support groups. Twelve semi-structured interviews with PwP and two group interviews, one with family members (*n* = 4) and one with physiotherapists (*n* = 5), were conducted. Interview guides were informed by patient–public input and a recent systematic review. Interviews were recorded, transcribed, and analysed using thematic analysis informed by the Grounded Theory methodology. Four common themes emerged: (1) The value of an intrinsic connection with exercise, for which there are challenges. A greater connection to exercise led to long-term adherence. (2) Adapting exercise to the needs and preferences of a person is essential. Preferred exercises and environments were mixed, with differences emerging between sexes. (3) Physiotherapists’ aim to only maintain physical function led to frustration. Limited self-management opportunities, stigma, and dehumanisation were discussed. (4) Non-motor symptoms, stigma, fear, and determination as well as apathy, pain, and low mood were discussed. Exercise provided physical, emotional, and social rewards. Supports are necessary; however, challenges arise when PwP’s motivations are mismatched to family members’ and physiotherapists’ goals. Co-created goals, tailored to their preferences, and exercise plans with supported self-management are recommended.

## 1. Impact Statement

Despite considerable barriers to exercise, the PwP interviewed were committed to exercise to improve function. However, mismatches between PwP’s goals and the level of support provided by their family members and physiotherapists were common, leading to frustration. Co-created exercise plans and goals, adapted to the person’s preferences and needs, can help maintain sufficient exercise intensity and safety.

## 2. Implications for Rehabilitation

- Differences in exercise patterns and preferences between sexes should be considered when designing exercise programs (targeting individual’s intrinsic motivators and perceived needs);
- Family members are crucial in supporting exercise, and physiotherapists rely on them, especially in cognitive decline. However, frustration can arise when they “push” exercise rather than supporting exercise;
- Explaining the rationale for tailored exercises is important, but people value having ownership and independent self-management of their exercise programs;
- Exercise intensity is often under prescribed, missing the opportunity to guide people to high-intensity exercise, which may have a neuroprotective effect, slowing disease progression;
- Highly functioning, motivated people feel excluded from challenging exercise. They are signposted to community-based classes or neurological gyms, where they feel underchallenged.

## 3. Introduction

Alongside pharmacological management, exercise is important in the management of Parkinson’s Disease. Exercise has been shown to attenuate motor and non-motor symptoms [1] and improve sleep [2], mood [3], and memory [3]. There are some indications that exercise can subsequently slow disease progression, especially in the early stages [4,5]. The positive effects are detected across a wide spectrum of exercise types and intensities; high-intensity aerobic exercise, high-intensity balance training, and unstructured physical activity have all shown attenuated motor symptoms and improved function [6], irrespective of nigrostriatal degeneration [7].

However, adherence to exercise is challenging. Despite the growing evidence of its value, as few as 30% of people with Parkinson’s (PwP) with mild disability achieve the World Health Organisation recommended activity levels [8,9], with some inactive for 70% of the day, which is considerably less active than age-matched peers [10]. Ellis et al. [11] identified self-efficacy and outcome expectations as strong predictors of exercise adherence in PwP. Similarly, Dobkin [12] recommended self-management strategies in all neurological rehabilitation to improve exercise adherence, optimise motor learning, and improve long-term outcomes. A recent systematic review examined the effects of behavioural change (BC) interventions to improve exercise self-efficacy and adherence in PwP [13]. The BC interventions were mapped to the Theoretical Domains Framework [14], which helped identify effective core components across the interventions [14]. Most were small exploratory studies [15,16,17,18,19,20,21], and interventions were multicomponent [15,16,17,18,19,22], including education, behavioural techniques, and support groups. While exercise self-efficacy or adherence remained unchanged, it appears that QoL and physical function improved [11]. The most effective TDF domains were behavioural regulation, belief about capabilities, social influences, reinforcement, and goals [13]. Heterogeneity between studies prevented a meta-analysis, highlighting the novelty of the topic and need for further exploration [13].

While self-efficacy and exercise adherence seem to be difficult to change, qualitative evidence suggests that the social and physical environment [23,24] and family support [23,24,25] are important in sustaining physical activity. In light of this, we need to explore family support and physiotherapy support in sustaining physical activity. Early physiotherapy involvement, daily exercise, and support from both clinicians and family empower PwP to stay motivated and involved in activities [26]. Many PwP look forward to engaging with their therapists, exercise instructors, and fellow classes members [27]. While many studies have explored barriers and enablers of PwP, they have not provided insight into their support systems, i.e., their physiotherapists’ and family members’ experience of supporting exercise and activity to PwP.

Therefore, this study aims to explore motivation, support, and self-management to keep physically active among PwP and to discuss these further with family members and physiotherapists. Interviewing PwP, family members, and physiotherapists provides us with an understanding of PwP’s motivation to exercise and the challenges to supporting meaningful, enjoyable exercise for all involved. To gain insight into the relationship between their experiences, this is a sequential qualitative interview study with the first phase informing the subsequent phases. In Phase 1, through semi-structured one-on-one interviews with PwP, the main supports and motivational needs of the PwP were identified. These topics were brought to the subsequent Phase 2 group interview with their family members and Phase 3 group interview with their physiotherapists.

The findings herein will inform a behavioural change intervention to improve exercise self-efficacy and exercise adherence in PwP.

## 4. Methods

### 4.1. Design and Ethical Considerations

The study was conducted between November 2022 and January 2023 and is reported following the consolidated criteria for reporting qualitative research (COREQ) [28]. Local ethical approval was received (ECM 4 (d) 11/1/2022).

### 4.2. Recruitment and Study Sample

Purposeful and maximum-variation sampling methods were employed to recruit PwP of varying age, sex, geographical setting, and disease severity. Posters were used in urban primary care centres and local support groups to recruit participants and their family members. Interviews were offered in person, by phone, and online. People with cognitive and mood disorders were not excluded from the study; it was suggested that a family member attended (dyad interview) to validate the data. A convenience sample of local community/hospital (public healthcare) physiotherapists with a minimum of three years’ experience of working with PwP was recruited by email.

### 4.3. Data Collection

The topic guides were informed by a recent systematic review [13], clinical experience, and patient and public involvement group (PPI) (see Supplemental Data). In line with this systematic review, the Theoretical Domains Framework was used to structure the topic guide to explore barriers to exercise [14], focusing on the five key domains of behavioural regulation, belief about capabilities, social influences, reinforcement, and goals [12].

The interviews were conducted by a registered physiotherapist (with qualitative research training. All interviews lasted up to one hour and were conducted in a quiet, private room at the physiotherapy department. At the end of the interviews, all participants briefly reviewed the interview for verification. Between interviews, the data were checked for emerging themes and to allow self-reflection. Phase 1 was terminated when data saturation based on code meaning [29] (no new themes emerging and data beginning to repeat),was reached. All interviews were audio-recorded, transcribed, and checked for verification. The transcripts were used for analysis.

### 4.4. Data Analysis

The data were analysed using a Grounded Theory approach, using Braun and Clarke’s reflexive thematic analysis [30]: (1) The data were reviewed with reflexivity, highlighting the key notes. (2) The text was coded, and highlighted data were extracted. Codes were reviewed by a second experienced qualitative researcher. An integrated code and corresponding list of quotations was completed. (3) Subthemes and themes were developed. (4) The themes were independently reviewed for overlap and sense-making. (5) Refinements were made, similar topics were merged, and large topics were split repeatedly until all authors reached a consensus, with no new themes. (6) Finally, all authors examined the relationship between the themes and finalised the report with supporting illustrations.

## 5. Results

### 5.1. Participants

Forty-two people were contacted (*n* = 20 PwP, *n* = 12 family members, and *n* = 10 physiotherapists); twenty-one were interviewed, (*n* = 12 PwP (6 women), *n* = 4 family members (1 woman), and *n* = 5 physiotherapists (5 women)) (see Table 1). Two PwP completed a dyad interview: One was online, and one interview was completed by telephone. Neither dyad-interviewed family member participated in the family member group interview. All physiotherapists were at senior grade (Table 1). One person with Parkinson’s engaged in advanced therapies (deep brain stimulation). Eleven PwP self-reported that they engaged in regular physical activity; the twelfth self-reported that they exercised infrequently. Eight were urban dwellers, while four were rural dwellers.

### 5.2. Theme 1: The Value of an Intrinsic Connection with Exercise: But There Are Challenges

A common consensus among people with Parkinson’s was that exercise was wider than just planned or structured activity. An intrinsic connection with exercise (an extremely important and basic characteristic of a person; being closely connected with exercise) was linked with long-term exercise adherence. Those with a deeper connection viewed it as a relationship rather than a task (“It’s freedom”), and while personal, it was portrayed as a pleasant experience providing physical and mental benefits (*P1:* “It invigorates me, it makes me feel brave, it lifts me”; “It’s just my time and my space”). Overall, it appeared that those intrinsically motivated to be active prior to diagnosis displayed stronger exercise self-efficacy and self-motivation, which are important predictors of long-term adherence. Importantly, exercise seemed to provide a sense of control and empowerment, bringing greater beliefs about their capabilities, especially that exercise was essential for “fighting” Parkinson’s.

Family members understood its value and often attempted to motivate PwP to exercise. Sometimes their attempts worked, but other times, it led to disagreements, thus leaving them reluctant to suggest exercise. Some described the emotional burden, as they felt pushed by health professionals to encourage exercises rather than supporting the PwP’s intrinsic motivation to exercise (*FM1:* “Tom gets irritated with me, I have, you know all the [exercise] posters up… he gets mad, says I’m nagging him”). Similarly, physiotherapists acknowledged the value of family to support and promote exercise (*PT1: “…* I think it’s really important that they’re involved in the whole… physio treatment with the patient”) and relied on family members, especially during cognitive decline (*PT5:* “They might need prompts… the carers are kind, of your source of information”). Ultimately, the turning point from supporting to pushing is to be respected. This finding suggests that family members can be guided to give support and companionship in exercise rather than rely on them to be the (external) motivator at home.

The analysis of the transcripts was underpinned by Grounded Theory methodology to better understand the meanings of participants’ subjective experiences with engaging in exercise and physical activity, their supports, and social actions. Data analysis led to four themes across, family members, and physiotherapists. Each theme is presented as PwP’s perspectives, then family members’ perspectives, and finally physiotherapists’ insights. The subthemes and representing quotes are displayed in Table A1 (see Appendix A).

### 5.3. Theme 2: Adapting Exercise to the Needs and Preferences of a Person Is Essential

Most PwP felt that exercise should be fun and enjoyable; if personalised, exercise was viewed as more appealing and effective. People were more motivated to embed simple exercise into daily life (*P1:* “… when you are standing at the sink lift your legs, move them up and down”; *P5:* “Walking it’s movement like- it’s not all about the machines, like”).

All the family members understood that exercise was important (*FM3:* “… last year… [I] bought the bike… The greatest thing I ever bought for exercise, and she uses it. That’s if she can’t get out on a day like today… So, I think exercise is paramount”; FM1: “If I can get my husband just even walking. So, he’s not sitting in his chair all day”). Physiotherapists agreed, stressing the importance of accessible exercises easily embedded into daily lives (*PT2:* “To try and make this a more normal part of their lives”) and adapting the exercises to the patient’s ability and environment.

Discussions revealed diverse exercise preferences and settings for exercise among PwP. Many were motivated to exercise outdoors (*P4:* “We need to keep fit, and we need to keep outdoors”) while others liked a local supervised gym with fully accessible equipment (*P5:* “You are around other people with… similar ailments like stroke”). Being able to exercise created a sense of belonging and support among PwP, promoting community engagement.

Men and women’s opinions about exercising in groups were different. All the women appreciated the peer support of a sociable group. Conversely, men either stated no preference or preferred to exercise alone, suggesting stronger intrinsic motivation. The benefit from socialising through exercise was also expressed by the family members, while this did not come up in the physiotherapists’ group interview. Four women discussed the value of mindfulness, describing how it helped self-manage their condition by allowing a deeper physical and mental connection with exercise, supporting long-term adherence (*PT 4:* “I started looking into mindfulness, and I then realized… I didn’t even know my body”).

These findings revealed that all participants value exercise. Taking time to consider unique exercise preferences could motivate engagement and enjoyment of exercise.

### 5.4. Theme 3: Physiotherapists’ Aim to Only Maintain Physical Function Led to Frustration (This Theme Was Not Expressed by Family Members)

Conversations revealed frustration and dissatisfaction amongst people with Parkinson’s when the support they receive does not match their needs. While people were motivated to improve function through exercise, they were frustrated with the physiotherapists’ primary goal to maintain function. Many PwP felt that physiotherapists were overly authoritative rather than supportive.

Those with fewer motor symptoms felt particularly excluded by physiotherapy services. They were rarely offered physiotherapy but rather were signposted to other community-based exercise classes, which did not seem to be a good fit for their needs. At most, they were offered physiotherapy-led exercise classes, where they felt that their condition of Parkinson’s was seen rather than them as individuals and their broader needs. They perceived that the exercises’ dosage and intensity were too low to be effective. A community-based gym (providing accessible equipment, physiotherapy prescribed programs, and supervision) was offered frequently where they again felt underchallenged, or exercises did not progress sufficiently to be challenging (*P3:* “But why can’t exercise help me get better; why did it have to be at best a flat liner?”). A common assumption was that physiotherapists were overly concerned about potential adverse events with exercise progression.

Most PwP wanted to have some control and responsibility of their own exercise programs, with the freedom to progress and adapt exercises with the guidance of the physiotherapists. They wanted to learn how to exercise with the view of developing self-management and independence.

Physiotherapists too expressed frustration. They believed they need to educate people how to exercise safely and correctly in the early stages and motivate people to exercise. Equally, the physiotherapists described that some PwP need a lot of education to even begin exercising. Physiotherapists’ discussions showed that their focus is to maintain function as the disease progresses, conflicting with the PwP’s motivation to improve rather than maintain function. Resource issues frustrated the physiotherapists: lack of accessibility, staffing, and expert knowledge among available staff. One physiotherapist recognised these high-functioning individuals were not being referred to physiotherapy as well as resource issues. Family members did not voice any concerns about physiotherapy services—response bias may have influenced these findings.

These findings revealed that people were motivated to exercise to improve and to be more responsible for their own exercise programs. Conversely, physiotherapists felt their primary obligation was to keep people active rather than to progress exercises and were frustrated with resources.

### 5.5. Theme 4: Non-Motor Symptoms, Stigma, Fear, and Determination

People with Parkinson’s described how the non-motor features associated with Parkinson’s including pain, fatigue, apathy, cognition, and sleep disturbance prevented exercise considerably. Family members and physiotherapists were aware of this phenomenon also.

Stigma was a real issue cited by people with Parkinson’s. (*P1:* “I’m walking in and I’m thinking the whole place is looking at me”; “I don’t go out for people to see me walking”). A feeling of “being a burden” was common when PwP needed support to exercise.

Conversely, anxiety, fear of progression, and a determination to stay well and independent were strong motivators to exercise. Some people with Parkinson’s and their family members spoke about “not giving up” (*P1:* “I can’t give up…”; P3: “… refusing to give in”) or were more explicit in the perceived “fight” against Parkinson’s Disease, while others spoke more in terms of maintaining function.

Physiotherapists recognised additional barriers to exercise, including fear of falling/injury non-motor symptoms such as mood (*PT4:* “The apathy, the depression like all that that can exist in the absence of… motor problems”) and transport.

## 6. Discussion

This study aimed to explore motivation, support, and self-management to keep physically active among people with Parkinson’s and to discuss these further with family members and physiotherapists. Four themes emerged:

- *An intrinsic connection with exercise provides physical and mental benefits, which family and physiotherapists are keen to support, but it can be challenging when they are mismatched;*
- *Adapting exercise to the needs and preferences of a person is essential;*
- *Individuals were frustrated by physiotherapists’ lack of exercise progression and guidance on self-management, while physiotherapists were frustrated with resource issues;*
- *Non-motor symptoms and social stigma are considerable barriers, while fear and determination are motivators to exercise.*

Many participants agreed across themes: the value of exercise in Parkinson’s, the need for accessible exercise, and non-motor symptoms as a barrier to exercise. However, some conflicting opinions arose around the motivation to exercise, to improve function, to “fight” Parkinson’s (PwP), or to maintain function or prevent deterioration. There was frustration among PwP who were motivated to exercise more intensely and among physiotherapists who prioritised safe exercising and fair use of limited resources.

This research identified three key considerations for exercise services for PwP. These include careful tailoring in exercise prescription, including sex considerations; the importance of creating shared responsibility for exercise progression; and preventing the exclusion of higher-functioning PwP in exercise classes. Although data saturation was achieved in this high-functioning cohort, there is a need to explore whether these findings/issues are similar in people with limited function.

### 6.1. Considering Sex in Tailoring Exercise Prescription

Sex-specific exercise preferences in this study included types of motivation, the ideal exercise environment, and mindfulness. Women with Parkinson’s appeared to be motivated to exercise with peer support and company, while men with Parkinson’s engaged in exercise for the physical and/or mental benefits. Family members also valued the social aspect of exercise for the PwP. van Uffelen et al. [31] similarly found that older women were more motivated by spending time with others, meeting friends, or improving appearance, while older men were motivated by managing stress, preventing health problems, and the “feel good” factor associated with exercise [31].

Women consistently reported enjoying exercising outdoors, while men did not express a preference. However, this does not seem to be universal. Older men were found to prefer outdoor activities more compared to older women [31,32] and described it as a “spirit lifter” [33] (p. 9). Generally, outdoor activity was preferred among both men and women Swedish and Irish seniors [34,35]. The importance of being outdoors with nature was highlighted in this study.

Other “spirit lifters” mentioned were music, enjoyment, fun, and mindfulness. Mindfulness motivated some women to self-manage and adhere to exercise; this was not the case among men. Blair Kennedy et al. reported improved exercise adherence and self-efficacy when mindfulness is integrated with exercise [36]. As an emerging area of research, Nymberg et al. [37] in Sweden are currently examining the impact of mindfulness on motivation and adherence to physical activity in middle-aged to older adults [37].

### 6.2. Shared Responsibility to Tailor Exercise Type, Dosage, and Intensity

Importantly, PwP want to share responsibility for their exercise progression. Patient-centred care is at the forefront of good practice [38]; however, when prescribing exercises, this principle can be inadvertently de-prioritized. PwP felt unheard and frustrated with little shared ownership of their exercise programs.

Noting that the interviewees were active exercisers, the PwP believed that they were capable of more challenging exercise, which they felt was largely overlooked. Instead, they perceived that physiotherapists were afraid to progress exercises and that exercises were prescribed “for the Parkinson’s Disease” rather than for the person (physical and mental health), potentially missing the opportunity to support high-intensity, potentially neuroprotective exercise. People lacked a sense of belonging when they were directed away from physiotherapy to community-based classes or gyms, leaving them feeling disengaged from physiotherapy and their Parkinson’s community. These findings highlight the need for patient-led, tailored exercise adapted and progressed to cater for the individual and mindful of the wider social, physical, and mental health benefits of exercise and its settings.

Exercise can improve balance, strength, gait, and physical function [39] and non-motor features including quality of life and depression [40]. Emerging evidence suggests that high-intensity exercise (65–85% max. heartrate) is associated with neuroprotective effects, slowing disease progression, and appears to be feasible and safe [6], but more research is required. Thus, healthcare professionals should consider higher-intensity exercise initially for PwP and keep informed of emerging evidence.

The mismatch between the goals of the physiotherapists and PwP through exercise emerged by using the sequential interviewing method and focused our exploration on the interviews. Initially, “under prescribing exercise” was identified as important to PwP from their interviews, which then informed the topic guide for the physiotherapists’ interview group.

### 6.3. Strengths and Limitations

A few factors hinder the transferability of the findings. Firstly, the study mostly represents high-functioning PwP. Although the recommendations provided by Mutrie et al. [41] were followed, and every attempt was made to recruit people with varying exercise adherence, the sample included participants who were more interested/already engaged in exercising only. Secondly, the sample size of PwP is small [42]; however, guided by information power versus data saturation [43], rich data from twelve high-functioning PwP with varying sex, years since diagnosis, and living circumstances were gathered. Data saturation was achieved with no new emerging themes in the final interviews. Furthermore, the small sample size of both interview groups limits the transferability of this study. For future studies, we would like to include a larger sample of family members and physiotherapists. Finally, the findings only represent physiotherapists who worked in the Irish National Public Health system. All were women; recruiting male physiotherapists was challenging, as 74% of physiotherapists in Ireland are women [44].

The interviewer (L.A.) is a registered physiotherapist, whose special interest and clinical expertise in Parkinson’s, which may have led to better iterative questioning and credibility. While all participants were encouraged to be open and honest during the interviews, an existing physiotherapist–patient relationship between the interviewer and PwP and the family members may have affected open dialogue and participant responses. Every attempt was made to minimise this, including keeping the conversation on “exercise” rather than physiotherapy services. Data analysis was completed by three team members, improving credibility, and the interviewer completed a reflective diary between interviews.

## 7. Summary and Key Messages for PwP, Clinicians, and Family Members

The interviews highlighted an interest from all parties to willingly partake in health and activity. However, a discordance emerged at times between the person with Parkinson’s and their supportive network, leading to frustration and challenges. It was evident that family members were frustrated if PwP were not active “enough”. Physiotherapists were frustrated with resource issues, and PwP were frustrated with the lack of support and focus on maintaining function from physiotherapy services. Ultimately, the interviews highlighted the need for a patient-centred, patient-led approach to support, beginning at a level they feel comfortable with.

The data showed the importance for PwP to express their motivation and preferences for exercise. Factors such as social engagement and mindfulness were important, especially among the women interviewed, while men expressed frustration with exercise prescription being “a flat liner”. Rather than encouraging more exercise, family members could explore motivation and personal preferences to identify meaningful ways to exercise or stay active, such as the stationary bike being “the greatest thing [they] ever bought” or “being out with other people”. Importantly, physiotherapists need to hear the PwP’s preferences and their challenges and guide family members to be supportive in the home rather than being “coaches”. Through hearing PwP motivation and preferences, a meaningful and engaging program can be co-created. Opportunities to exercise beyond physiotherapy can be explored. It is important for the individual to be safe and enjoy the activity.

Opportunities to self-manage were often overlooked. It was evident that PwP were well informed; however, the service demanded a crutch-like dependency on physiotherapy guidance, with limited resources. Parkinson’s Disease is a lifelong condition, and providing all PwP with the skills and knowledge to self-manage their condition is important, empowering, and respectful. Education, signposting to services, safe exercise guidance, highlighting issues that may need medical attention, and ease of access to services when required will facilitate self-management.

It is important to change and adapt exercise routines. As guided by the evidence, including strength, balance, aerobic, and stretching in all exercise routines is recommended. The intensity must be adequate to ensure effectiveness. More specific exercises should be included to target individual challenges, i.e., hip or knee strengthening for pain. As noted, exercise intensity is often under prescribed. To prevent this, exercise intensity should be monitored, for example, by the Rate of Perceived Exertion Scale [45].

Suitable outcome measures can include validated physical or functional performance measures in Parkinson’s (six-minute walk test; 30 s sit to stand) and a self-reported QoL measure (PDQ-39). Qualitative indicators of effectiveness can include self-reported motivation and adherence and family members’ perceptions.

## 8. Conclusions

To our knowledge, this is the first study exploring motivation, support, and exercise self-management needs in Parkinson’s Disease through multiple viewpoints. An important finding is the mismatch between physiotherapist and PwP goals with exercise. Physiotherapists were perceived to focus on the condition rather than the physical, social, and mental wellbeing of the individual. Higher-intensity exercises were sought by PwP, and early evidence suggests that it can be neuroprotective and safe. Exercise preferences appeared different between sexes and could be considered to make exercise more enjoyable. Women were more motivated by external factors such as social interaction, while men had higher intrinsic motivation to exercise. Most importantly, this study highlights the welcome of a shared responsibility between therapist and patient as part of the overall disease self-management. Family members play an important role in supporting exercise, but an expectation to drive or coach exercise can be detrimental. Although this study included a small sample size and has a low representativeness of the patients enrolled across the disease spectrum, the findings of this study provide insights for improving clinical practice and future investigations on this topic.

## Figures and Tables

**Table 1:** Clinical characteristics of participants.

Phase 1 Participants: People with Parkinson’s									
Code	Interview performance method	Age (years)	Sex (M/F)	Hoehn and Yahr Stage	Years Since Diagnosis	Geographical setting	Mobility	Activities of Daily Living	Main Caregiver
P1	In person	57	F	1	4	Rural	Independent (no aid)	Independent	Oneself
P2	Phone interview	-	F	3	12	Urban	Independent (no aid)	Minimum assistance	Oneself
P3	In person	57	M	1	17	Urban	Independent (no aid)	Independent	Oneself
P4	In person	62	F	1	2	Urban	Independent (no aid)	Independent	Oneself
P5	Online dyad interview (spouse present)	68	M	2	6	Rural	Independent (with walking aid)	Moderate assistance	Spouse
P6	In person	52	F	1	2	Urban	Independent (no aid)	Independent	Oneself
P7	In person	84	M	2	8	Urban	Independent (no aid)	Independent	Daughter
P8	In person	82	M	2	11	Urban	Independent (with walking aid)	Minimum assistance	Spouse
P9	In person	76	F	3	6	Rural	Independent (no aid)	Moderate assistance	Spouse
P10	In person	73	F	2	8	Urban	Independent (no aid)	Minimum assistance	Oneself
P11	In person	73	M	1	1	Rural	Independent (no aid)	Independent	Spouse
P12	In person dyad interview (spouse present)	76	M	3	6	Urban	Independent (no aid)	Independent	Spouse
**Phase 2 Participants: Group interview with family members (of participants from phase 1)**									
Code	Sex	Relationship with phase 1 participant				Phase 1 participant code			
FM1	F	Spouse				P8 (11 years diagnosed)			
FM2	M	Spouse				P4 (2 years diagnosed)			
FM3	M	Spouse				P10 (8 years diagnosed)			
FM4	M	Spouse				P9 (6 years diagnosed)			
**Phase 3 Participants: Group interview with physiotherapists**									
Code	Sex	Years working years with PwP				Work Location			
PT1	F	14				Residential care			
PT2	F	7				Urban primary care			
PT3	F	5				Urban primary care			
PT4	F	15				Outpatient department			
PT5	F	12				Rural primary care			

## Data Availability

The datasets presented in this article are not readily available to protect participant confidentiality. Requests to access anonymised partial datasets should be directed to r.mccullagh@ucc.ie.

## Supplementary Materials

The following supporting information can be downloaded at: https://www.mdpi.com/article/10.3390/geriatrics9040092/s1. Section SA1: Interview Questions—Semi-structured interviews: People with Parkinson’s. Section S1B: Interview Questions—Group Interview 1: Family-Member/Carers. Section S1C: Interview Questions—Group Interview 2: Physiotherapists.

## Appendix A

**Table A1:** Themes, Subthemes, and Quotations.

Theme 1: The value of an intrinsic connection with exercise: but there are challenges.	
People with Parkinson’s	
**Subtheme**	Quotation
**Meaning of exercise**	P3: Exercise is anything and everything you know, it can literally be a walk to the shops, getting up and walking up and down the stairs.
	P9: … as long as you are moving its exercise.
**Physical and mental benefits of exercise**	P11: I believe a good workout sorts a lot of other problems. It flushes out the system, it rejuvenates it. If I don’t get out for three or four days with three or four weeks, my mental health and everything starts and slide.
**Exercise is essential to “fighting” Parkinson’s**	P3: I would try to combat it [Parkinson’s] through exercise. Doing the exercises isn’t necessarily for fitness, it’s about not letting it take control over you. You’re both there under sparing of each other, who’s going to win? Who’s gonna last the longest?
	P2: I’m still able to do the things I want to do, and I believe it’s just because I exercise regularly.
**Intrinsic motivation leads to long-term adherence**	P11 (male): … I always like to exercise; I think it is just a gene you are born with.
	P4 (female): I always prided myself on having the insight to know that we need to keep fit.
**Family members**	
**Subtheme**	Quotation
**Reluctance to suggest exercise**	FM3: It depends. She’s always saying, I’m tired and I have something else to do. And she’ll always find an excuse.
	FM4: I had it out with Julie three weeks ago, four weeks ago… Because I felt that, I was taking over all that and I suppose, you see, if [she is] the recipient of that, [she] can say “I’ll sit back for a session, he is going to remind me to do those things”.
	FM1: Tom gets irritated with me; he gets mad says I’m nagging him.
**Physiotherapists**	
**Subtheme**	Quotation
**Emotional burden on family members**	PT1: Yeah, I agree. I guess over the years I felt sometimes not like a referee… and then the wife’s like ‘I’m always telling him that.’ And they like ‘he gets cross when I remind him’ you know.
**Theme 2: Adapting exercise is essential.**	
**People with Parkinson’s**	
**Subtheme**	Quotation
**Exercise should be personalised and adapted to each individual**	P11: Well, what works for me doesn’t work for a lot of people. But there is a lot more to it than that you have to check the individual and design a program that suits him.
**Exercise preferences varied per individual**	P4: I would have always been a very outdoorsy person… So, like, if the sun is shining… natural vitamins that are out there.
	P1: People with Parkinson’s should be outside; they should be outside and getting the natural vitamins from the sunlight rather than kept indoors and dark places.
**Group exercise created a sense of belonging**	P11: camaraderie is a great thing if, if you can inject, you can inject that into your group. They, they help each other then and it’s, it’s comforting.
**Exercise preferences varied among sex**	P2 (female): Even though the exercise may not be enjoyable, that social event is very important to keeping people going… These social events should allow for a social discussion. Don’t make it medical.
	P4 (female): I find I like to exercise in the group setting like a, so I don’t really fancy the walking. I walk with anybody. But I don’t like doing it on my own. And it just being part of a group.
	P10 (female): There’s good camaraderie… In the group. That are in there with me and… The craic, as they say (giggles). It’s the social aspect of it that’s it.
	P3 (male): It’s not the kind of thing that I would enjoy (group classes), and I think there’s a degree that you can put yourself under too much pressure to participate you know? I prefer to do it myself.
	P12 (male): [For] someone who needs it to be fun, I think group exercise would work. But I am happy exercising just me.
**Mindfulness**	P6: My joints were sore. Every part of me. Do you know? I know my body and I just knew, like, this is my Parkinson’s. So just take a break this week.
	P4: And, I started looking into mindfulness, and I then realized it’s so important to have a plan, to be able to know, I didn’t even know my body.
**Family members**	
**Subtheme**	Quotation
**Benefit of social element**	FM4: She is very happy about that, and I think it’s as well the meeting other people.
	FM4: She needs to be mixing. She used to get into the car and visit her sister’s every Thursday night and a couple of jazz get a taxi home and that kind of thing, so I think when she is exercising the socialising in a group is good for her.
**Physiotherapists**	
**Subtheme**	Quotation
**Adapting exercise to the patient**	PT3: We have to look at what we’re actually giving the patient, taking time to go through that with them and I suppose finding out what works for them what doesn’t work for them.
	PT2: The other thing then would be, I, when they can’t access it, ‘well then what exercise can you access?’ And saying that anything is better than nothing.
**Theme 3: Physiotherapists’ aim to only maintain physical function led to frustration.**	
**People with Parkinson’s**	
**Subtheme**	Quotation
**Physiotherapists should be encouraging not authoritative**	P1: Just say “you know what you’re doing brilliant now, come back in a month time I know better you’ll be after improving, we’ll see now, I bet you will be better”, not “you must do this, and you must do that.”
**Under prescribing—intensity too low, lack of exercise progression**	P3: Being told what you can do and can’t do. People telling me that I shouldn’t do this then I shouldn’t do that or I need to slow down or I need to stop… if I can cycle nine kilometres there and I’m going to cycle nine kilometres back, but my chart says I’m only supposed to do so many minutes so I can’t do any more than that… like I find it frustrating.
	P1: I have asked and asked and asked and I’m not being assessed. I shouldn’t have to ask to be assessed I should be assessed naturally; my exercises should be progressed as I get better.
	P12: The classes aren’t hard enough for me. I’m not saying it is beneath me, don’t get me wrong, but I just need harder to be beneficial
**PwP want more responsibility, independence to progress**	P3: Being told what you can do and what you can’t do. And I’m constantly trying to break the system you know. People telling me that I shouldn’t do this then I shouldn’t do that, or I need to slow down, or I need to stop.
	P3: But I jump on it, and I hop straight up to level 18 because I’m capable of this, and then he (physiotherapist) has a hernia.
**Family members**	
**Subtheme**	Quotation
**Mismatch between need and the service provided**	FM4: She has [Parkinson’s Disease] for about 7 years, and slowly but surely the deterioration, even though [the doctor] don’t notice it (speaking to interviewer), because [they are] not living with the person. Her strength is quite good, but a dexterity is awful, she can’t open the tops of bottles. She can’t put on her bra. Like you said (speaks to FM1) [I’m] actually caring for the person… if I leave the house, I have to leave something for her to eat. And I wouldn’t want to be going out too much like (laughs), because again it’s the dexterity issue… but every time I mention it to a doctor, I’m told she is doing well… all because she can walk perfectly.
**Physiotherapists**	
**Subtheme**	Quotation
**Education is the first step**	PT1: But I think it’s taken the time to actually teach them the right way to exercise, teach them what this is and like, try and get them started on that and look into the barriers that might be there.
	PT2: It’s a cultural thing. And a health literacy thing as well. They might have never exercised a day in their lives. Now they are being told they have a engage in regular exercise—that’s difficult for anyone.
	PT4: If the GP hasn’t accessed the service, people could have never accessed the services nor seen a neurologist, and they haven’t been educated so they don’t actually know that exercise is good.
**Focus is to maintain function**	PT2: Introducing them to exercise and showing them and how they’re going to maintain themselves.
	PT4: I suppose we see a lot of them when in with early (stages). You know, they’ve just been diagnosed with Parkinson’s. And I suppose the, your education component would be huge in that. They might be functioning at a very high level but just to educate on the progression of Parkinson’s and the importance of exercising at every stage and you know, the problems associated with progression.
**Lack of resources and expertise, poor awareness**	PT5: It’s really hard to give patients the amount of time they need and maybe the number of sessions they need to set them up.
	PT1: Not every therapist will have expertise in the area.
	PT2: GPs not thinking I should get them into physio now, because they’re not bad, they are relatively high functioning… And I think it often boils down to resources as well because… if somebody’s functioning well, there’s no resource there to look at that prevention piece.
**Theme 4: Non-motor symptoms, stigma, fear, and determination.**	
**People with Parkinson’s**	
**Subtheme**	Quotation
**Non-motor barriers**	P11: Unfortunately, I had to stop because when you’re racing you to make some very quick decisions and I that side [thinking and concentration] just doesn’t work right.
	P5: Well, I think it’s, it’s important not to try and do your exercises when you’re tired. Because if you do, you’re more inclined to make mistakes or twisting the knee or, you know, like.
	P4: Anxiety, anxiety is a big one.
**Stigma**	P6: Well for me. People have said to me, God, you don’t look like a person to have Parkinson’s. And my answer is, what does a person look like that has Parkinson’s. Maybe in the elderly 70, 80 he would walk in frame and bent over their limbs and stuff, you know and. But for a young person, what? What’s a young person supposed to look like with Parkinson’s?
	P4: It’s just the fact that when I say (about diagnosis) they’re probably expecting me to be doddering over or whatever.
**“Being a burden”**	P2: But maybe people have had to stop driving for one reason or another. Asking for lifts can prevent people from going to classes. Feels that they are in the way. Not only are people asking for lifts but during the class the driver must wait for an hour outside.
**Exercise to fight against Parkinson’s**	P4: OK, I have to give up my body to this disease at some stage and give more than I ever want, but it would take me kicking and screaming.
**Exercise to maintain**	P12: I would seize up. You know now when I say seize up, I would be stiff and slow.
**Family members**	
**Subtheme**	Quotation
**Non-motor barriers**	FM1: He can do it (exercise). He can do it. But he’s suffering very badly from mood swings, and he did before and then it got a bit better, and he exercised a little bit more. Mood swings and mood swings are a terrible thing because you don’t know when you have them.
	FM4: She knows what to do but she finds it very frustrating that she can’t do those things.
