# Supplementary material for: “Exercise… to Me, It’s Freedom”: Motivation, Support, and Self-Management to Keep Physically Active with Parkinson’s Disease: A Qualitative Study
Source: Geriatrics (Basel). 2024 Jul 11;9(4):92. doi: 10.3390/geriatrics9040092 (PMC11270374; doi:10.3390/geriatrics9040092)
Supplement: Supplementary file 1 [file geriatrics-09-00092-s001.zip › geriatrics-3043341-supplementary.pdf]

Supplementary Data

Section S1A

Semi-structured interviews: People with Parkinson’s

The topic guide for these interviews was mapped to the 14 domains of the Theoretical Domains Framework (TDF)[12]. Items covered in this paper are in bold.

Overview

Confirm consent to conduct and record the interview

Introductions and expression of gratitude to the participants

Reiterating the aims of the study

Encourage individuals to be open and honest about their experiences and give as much information as possible

Participant to state name, age, years since diagnosis (first symptoms), and whether they are independent with ADLs and living with someone or alone.

Knowledge, Skills, and Memory, attention, and decision processes (TDF Domains 1, 2 and 10)

1. When you were told you had Parkinson’s/received your diagnosis did you receive adequate information regarding your diagnosis of Parkinson’s? How did this effect/influence you/your ability to manage your condition?

1a. Were you surprised? Can you tell me about that?

2. Has anyone talked to you about exercise and physical activity? Can you tell me about that? *Probing Question: Do you think exercise and physical activity is important for you? Why? Probe their understanding of why exercise is important – is it more on a well-being point of view? Or a neuroprotective point of view?*

3. What do you think are the most important things for you when managing your condition?

4. Have you been informed of the role of exercise in the management of Parkinson's? If so, by who? How did this impact your outlook?

5. Is exercise important to you? Can you explain why/why not?

6. Do you believe you benefit from exercise? Can you explain?

7. Are you aware of the World Health Organisation's (or any) guidelines on physical activity? Can you explain this?

Belief about capabilities, Optimism, and Belief about consequences (TDF Domains 4, 5 and 6)

8. What activities do you consider to be "exercise"?

9. Do you engage in exercise? If no, can you please explain why? If yes, how many times a week to you exercise? What type of exercise do you enjoy?

10. Do you prefer to exercise alone? With a family-member/friend? As part of a group?

11. Other than physically, do you believe you benefit from exercise/how does exercise benefit you? Can you explain?

Symptom Management

12. Can you discuss some Parkinson's symptoms (both motor and non-motor) you have experienced? How do you manage these symptoms?

13. Do your symptoms effect your participation in everyday life? Can you explain?

14. How does exercise effect your Parkinson's symptoms?

15. How do your symptoms effect your ability to exercise?

Reinforcement, Emotion and Behavioural regulation (TDF Domains 7, 13 and 14)

16. What motivates you to exercise? /What encourages you to engage in exercise?

17. What reduces your motivation?

18. How do you stay motivated (strategies)? What techniques do you use to stay motivated?

19. As healthcare professionals what can we offer you or others to help you stay motivated/improve your motivation?

Barriers and Facilitators

20. Do you find it hard to exercise? If so, why?

Probe both personal and environment factors

Probe both motor and non-motor

Follow up question: How do you overcome this?

21. Do you believe it is easy for you to engage in exercise services in the community? Can you explain?

22. How can we make exercise services more accessible for you?

Intentions and Goals (TDF Domains 8 and 9)

23. Why do you exercise?

Probe the benefits, social element

24. Do you set goals for yourself? Can you give an example?

25. Are you as physically active as you would like to be?

If no, why not? What can you do to change this?

Environmental context and resources, Social influences and Social role and Identity (TDF Domains 11, 12 and 3)

26. Do you feel supported by your family and friends? Can you explain?

27. Do you feel supported within your community? Can you explain?

28. Do you believe the people you encounter during your everyday life understand your Parkinson's specific needs? Can you explain/How does this effect you?

29. What are the general society beliefs regards Parkinson's? How does this effect you?

30. Do you belief society facilitates/provides for your Parkinson's specific needs?

31. How can we improve this/What can we do to improve this?

Conclude

32. Ask participant if there is anything else they would like to say/add

33. Thank them for their participation.

Section S1B

Interview Questions

Group Interview 1: Family-Member/Carers

The topic guide for these interviews was mapped to the 14 domains of the Theoretical Domains Framework (TDF)[12]. Items covered in this paper are in bold.

Overview

Confirm consent to conduct and record the focus group

Introductions and expression of gratitude to the participants

Reiterating the aims of the study

Encourage individuals to be open and honest about their experiences and give as much information as possible

Participant to state name, their family-member/patient's name, if they are a family member or carer and how long ago since their family-member was diagnosed

Introduction

1. What are your experiences of living/caring for someone with Parkinson's?

2. Does your family-member engage in exercise? Can you explain (type, duration, frequency)?

3. Do you believe your family-member benefits from/enjoys exercise? Can you explain?

4. How important is exercise to your family-member?

5. What is your role in helping your family-member to exercise/ Do you play a role in helping your family-member to exercise?

6. What are the strengths and weaknesses to your role in helping your family-member to exercise?

Knowledge and Skills (TDF Domains 1 and 2)

7. When your family-member received their diagnosis did you receive adequate information and education regarding the management of Parkinson’s?

8. Were you educated regarding the role of exercise in the management of Parkinson’s? If so, by who? *How did this influence your outlook?*

9. Is exercise important to your family-member? *Can you explain why/why not?*

10. Do you believe your family-member benefits from exercise? *Can you explain?*

11. What are your thoughts and understanding of the role of exercise in the management of Parkinson’s?

12. As healthcare professionals, what can we do to help?

13. Are you aware of the World Health Organisation’s guidelines on physical Activity? *Can you explain this?*

Reinforcement, Emotion and Behavioural Regulation (TDF Domains 7, 13 and 14)

14. What challenges does Parkinson’s bring to exercise? *How do you help overcome these? Probe personal and environment factors; motor and non-motor*

15. What are the barriers and possible solutions to helping your family-member to exercise? *How can we help?*

16. As healthcare professionals how can we help overcome these challenges?

17. Can you discuss some examples of motivational technique you find beneficial to use to encourage exercise?

Intentions and Goals (TDF Domains 8 and 9)

18. Does your family member set personal goal when it comes to exercise? *Can you explain?*

19. What role do you play in the goal setting process? *Can you explain?*

20. Do you think your family member is as physically active as they would like to be? *If no, why not? What can you do to help them change this?*

Environmental Context and resources, Social influences and Social role and Identity (TDF Domains 11, 12 and 3)

21. Do you believe it is easy for your family member to access exercise services in the community? Can you explain?

22. Do you feel your family-member is supported within your community? Can you explain?

23. What are the general society beliefs regarding Parkinson's? How does this influence your family- member?

24. Do you belief society facilitates for people with Parkinson's?

25. How can we improve this?

Conclude

26. Ask participants if there is anything else they would like to say/add

27. Thank them for their participation

Section S1C

Interview Questions

Group Interview 2: Physiotherapists

Overview

Confirm consent to conduct and record the focus group

Introductions and expression of gratitude to the participants

Reiterating the aims of the study

Encourage individuals to be open and honest about their experiences and give as much information as possible

Participant to state name and how many years they have been working with people with Parkinson's Disease

Introduction

1. What is your role in helping people with Parkinson’s to exercise?

2. What are the strengths and weaknesses of your role?

3. Can you describe your Parkinson’s patient cohort (age, disease severity, sex)?

4. Are your patients with Parkinson’s physically active? Can you give some examples? Is it the same for both men and women?

5. Are your patients meeting the World Health Organisation’s guidelines on physical activity? Can you give some examples? Is it the same for both men and women?

Barriers and Challenges

6. What are common challenges to exercise faced by people with Parkinson’s? Do these differ for men and women? Can you explain

Follow up question: How do you help overcome them?

7. What barriers do you find challenging to overcome?

8. Can you discuss solutions/strategies you have discovered or suggestions you may feel worthwhile?

Education

9. Do you believe people with Parkinson's are well educated regarding the benefits of exercise?

10. Does this influences their outlooks of disease disability?

11. As healthcare professionals how can we improve this?

Role of the family member

12. Does the family member play an important role with regards to exercise among your patients? Can you explain this?

13. Do you believe family members and carers are well educated/understand the benefits of exercise?

14. Do you encourage family members to attend physiotherapy sessions? Can you explain?

Services

15. Do you believe people with Parkinson's are well supported within the community?

16. What clinical services do you offer for people with Parkinson's? Can you explain? Is access to these services' dependent on referrals? Can people attend these classes without a referral from a healthcare professional?

17. Outside of referral dependent clinically-led services are you aware of community exercise services available to people with Parkinson's? Can you explain?

18. With the prevention versus treatment approach in mind, how can we improve accessibility to clinically-led exercise services for people with Parkinson's not currently accessing community services?

Present findings from PwP and Focus group with family member

19. Discuss the need for this service, accessibility, acceptability, feasibility

Conclude

20. Ask participants if there is anything else they would like to say/add

21. Thank them for their participation
